# Variations in melatonin levels in preterm and term human breast milk during the first month after delivery

**DOI:** 10.1038/s41598-019-54530-2

**Published:** 2019-11-29

**Authors:** Yishi Qin, Weiyang Shi, Jialu Zhuang, Yu Liu, Lili Tang, Jun Bu, Jianhua Sun, Fei Bei

**Affiliations:** Department of Neonatology, Shanghai Children’s Medical Center of Shanghai Jiao Tong University School of Medicine, Shanghai, 200127 China

**Keywords:** Nutrition, Paediatric research

## Abstract

The objectives of the present study were to examine the dynamic changes in breast milk melatonin throughout the course of lactation and to explore factors associated with changes in melatonin concentrations and rhythms in both preterm and term breast milk. Breast milk was collected sequentially at 03:00, 09:00, 15:00, and 21:00 in one day. Melatonin was analyzed in 392 breast milk samples from 98 healthy nursing mothers at 0 to 30 days postpartum. In both preterm and term breast milk, the melatonin concentration presented a circadian rhythm with the acrophase at around 03:00. Subgroup analysis showed the peak melatonin concentrations differed significantly across lactation stages, with the highest concentration in the colostrum, followed by transitional and mature breast milk. At 03:00, preterm breast milk had a higher concentration of melatonin than term breast milk in the colostrum (28.67 pg/mL *vs*. 25.31 pg/mL, p < 0.022), transitional breast milk (24.70 pg/mL *vs*. 22.55 pg/mL), and mature breast milk (22.37 pg/mL *vs*. 20.12 pg /mL). Further studies are warranted for their roles and significance on melatonin in breast milk in nutrition and metabolism of neonates.

## Introduction

Melatonin (N-acetyl-5-methoxytryptamine), an endogenously produced indoleamine, is secreted by the pineal gland and mainly present in external secretions, such as serum, saliva and breast milk^1–3^. Melatonin has a wide range of biological functions, including antioxidation, anti-inflammatory, anti-apoptosis, and immunomodulatory, as well as adjustment of sleep and circadian rhythm^4–6^. Importantly, clinical trials have demonstrated that roles of melatonin in the prevention of neonatal sepsis and children’s sleep disturbances^7,8^.

Melatonin in human milk exhibits a pronounced circadian rhythm, reaching high levels at night but undetectable amounts during the day^2^. This may suggest that the melatonin fluctuation in breast milk indicates the time of day to infants. Some studies have attributed longer sleep time in breastfed infants compared with that in formula-fed infants to melatonin in breast milk, as no melatonin is detected in formula^9^. Being a normal component and a multifunctional protein in human milk, melatonin may significantly contribute to better growth, development and long-term outcomes in infants, which are associated with breastfeeding. A few studies have shown that concentrations of melatonin in human milk vary widely^2,9–12^. But no data is available on the human milk melatonin (HMM) level at different lactation stages. In this study, we identified HMM levels and explored the effects of gestation and lactation stages.

## Results

### Characteristics of participants

As shown in Table 1, a total of 98 lactating women successfully provided breast milk samples, 32 (33%) were mothers of preterm infants and 66 (67%) were mothers of full-term infants. The mean gestational age and birth weight were 34.1 weeks, 1.78 kg in preterm group and 38.9 weeks, 3.23 kg in term group, respectively. Mothers of preterm infants had a higher rate of delivery by caesarian section (78.12%) compared with those of term infants (33.33%).

**Table 1:** Characteristics of study participants between the two gestational groups.

Variable	Preterm (N = 32)	Term (N = 66)	p-Value
Maternal age (years)^a^	30.48 ± 4.47^a^	30.61 ± 3.89	0.79
Gestational age (weeks)^a^	34.09 ± 1.81	38.88 ± 0.98	**<0.01**
Delivery type (c-section%)^c^	78.12%	33.33%	**<0.01**
Parity^d^	1.41 ± 0.50	1.35 ± 0.48	0.99
Infant’s gender (male%)^b^	43.75%	43.94%	0.99
Body weight of infants (kg)^1^	1.78 ± 0.45	3.23 ± 0.42	**<0.01**

Statistical methods used: ^a^Unpaired test. ^b^Pearson chi-squared and data dichotomized into groups of female and male. ^c^Pearson chi-squared test and data dichotomized into groups of vaginal delivery and caesarean section (c-section) delivery. ^d^Mann-Whitney U test.

^a^Mean ± SD (all such values). SD − standard deviation.

### HMM levels

Table 2 shows the concentration of melatonin in 392 breast milk samples. Comparing at four different time points during one day, we found that HMM levels were relatively low during daytime hours (09:00 and 15:00), and then increased at night (21:00), peaking at 03:00 (mean value was 23.5 pg/ml), which was consistent with the results of previous studies^2^.

**Table 2:** HMM level according to the time of day.

Time point	03:00	09:00	15:00	21:00
Breast milk (pg/mL)	23.49 ± 4.25^a^	3.27 ± 1.23	2.40 ± 1.04	6.81 ± 2.35

^a^Mean ± SD (all such values).

### Impact of lactation stage

**Figure 1:**
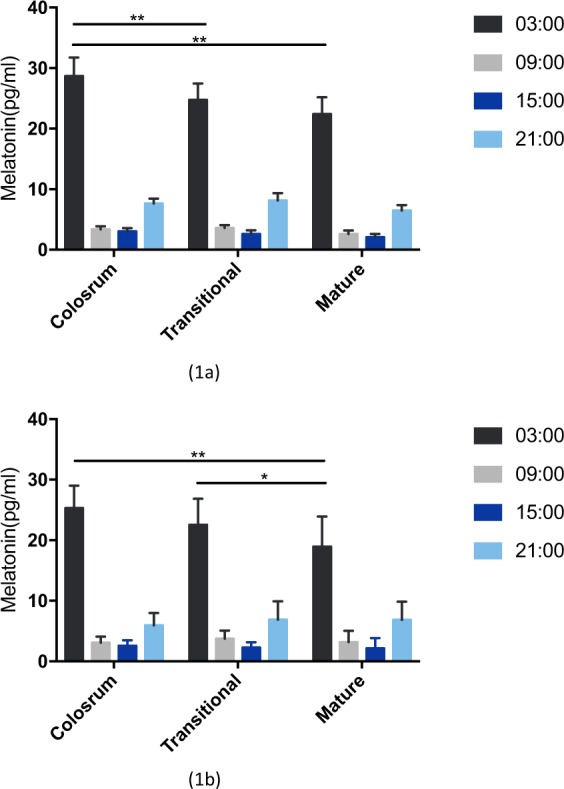
The melatonin level in preterm (**1a**) and term milk (**1b**) of different lactation stages. (*p < 0.05; **p < 0.01).

The melatonin concentration in breast milk of different lactation stages and collection times are presented in Fig. 1. There is a clear circadian rhythm of HMM level in preterm and term group, even in different stages of lactation. That means varying lactation stage shared a similar circadian rhythm of HMM, with a higher level in nighttime and a lower level during the day. Furthermore, with lactation prolonging the peak HMM level gradually decreased in both groups.

### Impact of preterm

**Figure 2:**
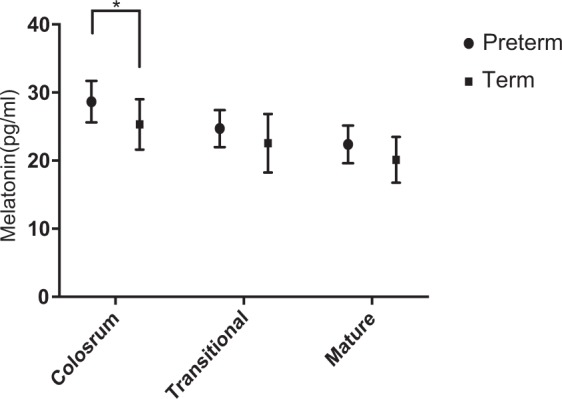
The peak HMM level of different lactation stages. (*p < 0.05).

In the current study, there was a trend of 3 pg/ml higher peak melatonin level in preterm milk than that of term milk across lactation. After stratification by stage of lactation, the mean peak melatonin concentration of colostrum was significantly greater in preterm human milk than that in term milk (28.67 pg/ml vs. 25.31 pg/ml, P = 0.022). However, no significant differences in melatonin concentrations between two groups in transitional milk and mature milk were observed (Fig. 2).

## Discussion

The present study characterized the melatonin level in preterm milk and term milk across different lactation stages. Understanding the levels and variations of HMM could contribute to the body of research that supports the importance of breastfeeding for infant nourishment and development.

In this study, for the first time, we demonstrated that melatonin has a clear circadian rhythm in both preterm and term breast milk across lactation stages. The current work identified that the peak melatonin level in breast milk was 23.49 pg/ml on average, which is comparable to previous data^2,11^. The peak HMM level accounts an average of 35% of the maternal serum concentration, which also shares a similar circadian rhythm^2^. That suggests HMM is likely derived from blood and the 24-hour HMM profile mimicked the melatonin levels changes in the blood^13^. HMM productions were relatively low during daytime hours, elevating at night, and then peaking at around 03:00, while artificial formulas do not contain melatonin^9,14^. Newborns have low serum melatonin levels due to an inadequacy of self-produced, which increase progressively up to the 3rd month of age when a characteristic circadian rhythm is first detectable^14^. HMM plays an important role in newborn synchronization with the mother’s rhythm^15^ and is responsible for longer sleep time in breastfed infants than that in formula-fed infants^9,16^. Some authors suggested that mothers should nurse with dimmer light or even lights off at night to avoid HMM reductions, which could disturb infant sleep patterns^17^. By the same token, differentiating milk pumped during the day from milk pumped during darkness has also been suggested for women that pump milk for their infants^11,18^.

In addition, we also found that HMM concentration changed dynamically during lactation in preterm or term breast milk. The peak HMM level was highest in colostrum, and then in transitional milk, and in mature milk, decreasing considerably during the first month after delivery. Honorio *et al*.^10^ found that higher colostrum melatonin levels at night appear to increase the phagocytic activity of colostral cells against bacteria. However, melatonin was shown to increase superoxide production and bactericidal activity of colostral phagocytes from normoglycemic women, but not from hyperglycemic women. This may suggest that the antioxidant effect of melatonin on the functional activity of colostral phagocytes decreased in diabetic women. However, limited data are available concerning the melatonin in the transitional and mature milk.

Compared with term milk, the preterm milk had a higher peak concentration of melatonin in every tested lactation stage, which may benefit for premature infants during the first few weeks after birth when they are especially vulnerable. Indeed, being a potential neuroprotective agent, melatonin was found to reduce brain injury and its long-lasting consequences after hypoxia-ischemia^19–25^ and oxidative damage in immature rat brains^26^. Furthermore, melatonin supplementation was also found to improve both lipopolysaccharide-induced neonatal inflammation and related brain injury in rats^27^, and the inflammatory reaction and cell death were reduced in the white matter of preterm and near-term fetal sheep following umbilical cord occlusion^28,29^. In view of undetectable plasma melatonin level before 31 weeks gestation age, an interesting proposed use of melatonin as a drug is to treat perinatal ischemic brain injuries^30^.

Our study had several limitations. There could be a selection bias. We attempted to diminish that by randomly approaching the mothers and explain the goal of the research only after they have agreed to cooperate. In addition, almost none of the mothers refused to participate. The small cohort size of preterm breast milk represented another limitation within our study. In the future, we plan to extend the breastfeeding period or divide the gestation ages into early preterm, middle preterm, and near term for more detailed research. Notwithstanding these limitations, this work offers valuable insight into the effects of preterm and lactation stage on HMM level, which provides an opportunity to develop hypotheses for future studies to evaluate how the HMM content was modulated on the basis of maternal and/ or infant factors.

This variability in HMM levels might reflect the different needs of different infants during the first few weeks of life. Our data might help to inform models to design tailored supplementation strategies of melatonin in nurseries and after home discharge. In fact, detection of HMM levels in specific mothers and during specific periods of lactation might reveal tailored melatonin supplementation for the infant according to the present maternal milk levels.

## Conclusion

This study has shown that melatonin has a clear circadian rhythm in both preterm and term breast milk across varying lactation stages. The peak HMM level was highest in colostrum, and then in transitional milk, and in mature milk, decreasing considerably during the first month after birth. Compared with term milk, the preterm milk had a higher peak concentration of melatonin, especially in the colostrum, which may benefit premature infants during early life when they are extremely vulnerable.

## Methods

### Study design

This cross-sectional study aims to evaluate whether there were differences of melatonin levels in the colostrum (0–7 days postpartum), transitional milk (8–14 days postpartum) and mature milk (15–30 days postpartum) for both premature milk and mature milk, and to explore the impact of gestational age on HMM.

### Subjects

Participants were recruited in the neonatal department and health care clinics, including mothers who delivered preterm infants (28–36 weeks gestation) or term infants (37–41 weeks gestation) from February to July in 2018. Infants with congenital malformations or dermatosis will be excluded. Some full-term and preterm infants have mild physiological jaundice with no other complications. The inclusion criteria were lactating mothers willing to comply with the study procedures. Any participants with chronic diseases (e.g., hypertension, diabetes mellitus, thyroid disorders, hepatitis, bronchial asthma, chronic renal failure, and heart failure), taking drugs (e.g., blockers and benzodiazepines) for sleep quality improvement, or suffering from acute infections, were excluded. Informed consents were obtained from all participants who volunteered to take part, along with breast milk samples. The investigations and human milk sample collections were conducted in accordance with the Declaration of Helsinki, and the protocol was approved by the Ethics Committee of Shanghai Children’s Medical Center affiliated with Shanghai Jiao Tong University School of Medicine, China (SCMCIRB-K2018069). All research was performed in accordance with relevant guidelines/regulations. Written informed consent was obtained by the patient or their legal representatives.

### Sample collection

We standardized sample collection procedures to facilitate sample’s consistency. HMM level at different lactation stages were individually donated sequentially at 03:00, 09:00, 15:00, and 21:00 in one day by mothers. Colostrum, transitional milk, and mature milk were defined as the milk within 0–7 days, 8–14 days, and more than 14 days postpartum, respectively. A complete milk expression from one breast was pumped into a feeding bottle and then 2 to 5 ml milk was transferred into breast milk bags and stored at −20 °C. Milk samples were subsequently transported in a cooler with ice to our central laboratory, where it was frozen to −80 °C until analysis. After defrosting the samples, milk was centrifuged at 12000 rpm for 10 minutes at 4 °C, then collected the middle layer ingredients and discarded the fat. The middle layer ingredients were tested with IBL Melatonin ELISA kit to measure the melatonin concentrations according to manufacturer’s instructions (Immuno Biological Laboratories, Hamburg, Germany)^2^.

### Statistical analyses

Melatonin analysis data are reported as the mean ± standard deviation (SD). The differences between the concentrations of HMM were calculated using IBM SPSS 24.0 (Armonk, NY, USA), for parametric data, using an unpaired t-test. The Mann–Whitney U test and Kruskal–Wallis H test were used for non-parametric data. The results were considered significant when the p-value was reported at less than 0.05.
